# Medical students’ pattern of self-directed learning prior to and during the coronavirus disease 2019 pandemic period and its implications for Free Open Access Meducation within the United Kingdom

**DOI:** 10.3352/jeehp.2021.18.5

**Published:** 2021-04-06

**Authors:** Jack Barton, Kathrine Sofia Rallis, Amber Elyse Corrigan, Ella Hubbard, Antonia Round, Greta Portone, Ashvin Kuri, Tien Tran, Yu Zhi Phuah, Katie Knight, Jonathan Round

**Affiliations:** 1 St George’s, University of London, London, UK; 2 Barts and The London School of Medicine and Dentistry, London, UK; 3 King’s College London, London, UK; 4 George Davies Centre, Medical School, University of Leicester, Leicester, UK; 5 Imperial College London, London, UK; 6 University College London, London, UK; 7 North Middlesex Hospital, London, UK; Hallym University, Korea

**Keywords:** Access to information, COVID-19, Learning, Medical students, United Kingdom

## Abstract

**Purpose:**

Self-directed learning (SDL) has been increasingly emphasized within medical education. However, little is known about the SDL resources medical students use. This study aimed to identify patterns in medical students’ SDL behaviors, their SDL resource choices, factors motivating these choices, and the potential impact of the coronavirus disease 2019 (COVID-19) pandemic on these variables.

**Methods:**

An online cross-sectional survey comprising multiple-choice, ranked, and free-text response questions were disseminated to medical students across all 41 UK medical schools between April and July 2020. Independent study hours and sources of study materials prior to and during the COVID-19 pandemic were compared. Motivational factors guiding resource choices and awareness of Free Open Access Meducation were also investigated.

**Results:**

The target sample was 75 students per medical school across a total of 41 medical schools within the United Kingdom (3,075 total students), and 1,564 responses were analyzed. University-provided information comprised the most commonly used component of independent study time, but a minority of total independent study time. Independent study time increased as a result of the COVID-19 pandemic (P<0.001). All sub-cohorts except males reported a significant increase in the use of resources such as free websites and question banks (P<0.05) and paid websites (P<0.05) as a result of the pandemic. Accessibility was the most influential factor guiding resource choice (Friedman’s μrank=3.97, P<0.001).

**Conclusion:**

The use of learning resources independent of university provision is increasing. Educators must ensure equitable access to such materials while supporting students in making informed choices regarding their independent study behaviors.

## Introduction

### Background/rationale

The proportion of medical education delivered in the form of face-to-face lectures and other more traditional methods is in decline, due in part to a growing emphasis on self-directed learning (SDL) and the increasing availability of online and remote learning resources [1,2]. The coronavirus disease 2019 (COVID-19) pandemic has seen a dramatic acceleration of this evolution, with many clinical placements suspended and medical schools forced to move swathes of content delivery online [3,4]. However, arguably the most rapid and exponential growth in remotely available medical educational content has been independent of formal teaching institutions.

Free Open Access Meducation (FOAM) refers to a variety of sources of remote, freely accessible medical education. Typically, FOAM modalities include blogs, podcasts, and social media, but the definition can be expanded to other formats such as free online question banks. The term traditionally refers to both the online availability of medical information and the community contributing to and engaging with such data [5]. FOAM use in postgraduate populations has increased exponentially over the last decade [6-8]. However, while numerous studies have explored undergraduate use of individual FOAM resources, the extent to which medical students are aware of the FOAM movement and the proportion of independent study time they spend engaging with FOAM have yet to be thoroughly investigated. There is a particular deficit in understanding how students select and use resources independent of those created or signposted by educational institutions.

One significant study in this area suggested that although in-person lecture attendance remains a key method of learning, remote resources such as online question banks now comprise a similar proportion of medical student study time [9]. Resource choices appear to be significantly influenced by the purpose of use (e.g., learning new concepts versus revising previously encountered topics), with resources such as question banks and hand-written notes being utilized to a much greater extent than face-to-face delivery closer to exam time [9]. Those resources correspond to the format of many FOAM resources, which are often designed according to well-evidenced principles of knowledge attainment and retention, such as formative testing and spaced repetition [10,11].

### Objectives

This large, cross-sectional, remotely-delivered study aimed to investigate patterns in medical students’ SDL behaviors, their SDL resource choices (including the use of FOAM), the factors motivating these choices, and the potential impact of the COVID-19 pandemic on these variables. Ultimately, we aimed to identify patterns of study behaviors in undergraduate medical students that educational institutions can use to help tailor teaching and curriculum design both during and after restrictions imposed by the COVID-19 pandemic.

## Methods

### Ethics statement

Ethical approval was sought and granted by the St. George’s, University of London Research Ethics Committee (REC reference 2020.0111). Informed consent was obtained in the first section of the online questionnaire. All participants consented to their anonymous data being used for this study and/or additional data analysis and publication in a peer-reviewed journal.

### Study design

We employed a national cross-sectional mixed survey using a range of multiple-choice, ranked, and free-text questions. This article was described according to the Strengthening the Reporting of Observational Studies in Epidemiology (STROBE) statement [12].

### Participants

In addition to the authors of this article, 29 medical student collaborators representing 33 different medical schools within the United Kingdom, were recruited and responsible for survey dissemination across all cohorts within their institute of study (Supplement 1). Collaborators were encouraged to advertise the survey for a minimum of 2 cycles, a minimum of 2 weeks apart. Methods of survey dissemination included formal university communication channels, newsletters, social media, and informal communication channels. Graduate and undergraduate medical students were invited to complete the online survey between April 30, 2020 and July 1, 2020.

### Setting

*Inclusion criteria and exclusion criteria*: Medical students were included attending a university within the United Kingdom and consenting to the use of anonymous data for this study and/or additional data analysis and publication via a peer-reviewed journal. Students were excluded currently undertaking a pause in their studies or an intercalated degree and involving in the design or planning of the study.

*Validity and reliability of the questionnaire*: A draft survey was designed by the named authors. Similar previous research in the area was considered, and additional questions were added, directly addressing motivational factors and knowledge of FOAM [9]. The national survey comprised 16 items, including a mix of multiple-choice, ranked, and free-text response questions relating to basic demographic information and SDL behaviors (Supplement 2). The survey was piloted in a small group of medical students before being reviewed and launched in order to ensure that the questions were unambiguous and the phrasing did not generate responder bias. No issues were raised during the initial pilot of the survey. Participants accessed the survey via a link distributed by the collaborators. The questionnaire items consisted of diverse forms, including multiple-choice, ranked, and free-text responses. Therefore, a formal reliability test could not be undertaken.

The link took respondents to a Microsoft Form, which included both the informed consent form and the survey questionnaire itself. Students and collaborators distributing the survey were blinded to previous responses.

### Variables

Independent study time according to group and study materials, frequency of use of study materials, factor determining students’ opinion of FOAM resources, and awareness of FOAM were variables.

### Study size

A *post hoc* power calculation was conducted using G*Power ver. 3.1.9.4 (Heinrich-Heine-Universität Düsseldorf, Düsseldorf, Germany; http://www.gpower.hhu.de/). For the Mann-Whitney U-test evaluating study time allocated pre- and post-COVID of independent group, the power was calculated at 0.8505. Input values were as follows: two tails; parent distribution, Lapace; effect size, 0.2; alpha error probability, 0.05; and sample size, 168 and 1,396 (graduate students vs. undergraduate students). Effect size was set as low arbitrarily. If the comparison was for gender, power was 0.9933 (471 men versus 1,080 women). Effect size was set as low arbitrarily

For Wilcoxon signed-rank test of paired association, power was 0.8779. Input values were as follows: two tails; parent distribution, Lapace; effect size, 0.2 alpha error probability, 0.05; and sample size, 164 (graduate students). Effect size was set as low arbitrarily. If sample sizes were 1,396 (graduate entry group), 471 (men), 1,080 (women), powers were 1.0, 0.9996, and 1.0.

### Qualitative variables

We asked respondents to report their understanding of the definition of FOAM, and later coded this variable dependent upon its consistency with the accepted definition [5].

### Statistical methods

The statistical analysis was completed using IBM SPSS Statistics software for Mac ver. 26.0.0 (IBM Corp., Armonk, NY, USA). Descriptive statistics were used to analyze student demographics and study behaviors. Following an assessment of the data distribution, the Mann-Whitney U-test was used for between-group comparisons and Wilcoxon signed-rank test for paired associations. The Friedman test was used to compare differences in factors determining students’ use of FOAM resources by ranking them in order of importance. The Cochran Q test was used to assess changes in learning provisions over time.

## Results

Raw response data are available from: Dataset 1.

### Descriptive data of participants

**Fig. 1.**
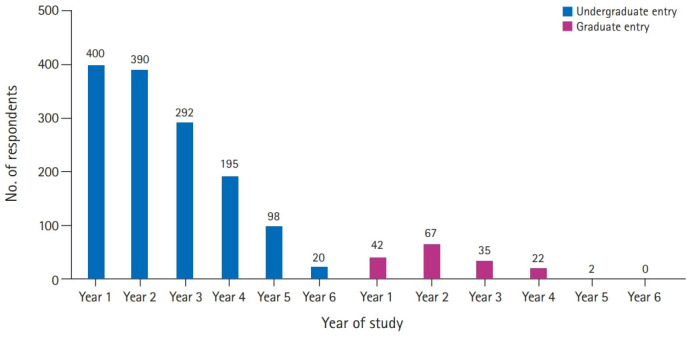
Year of study and MBBS (Bachelor of Medicine, Bachelor of Surgery) course details (undergraduate vs. graduate-entry program) of survey responders (n=1,564). One respondent (unspecified undergraduate) is not represented.

In total, 1,626 responses were collected, of which 1,564 were included in the analysis. Of the 62 responses excluded, 61 were duplicates, and 1 did not meet the inclusion criteria as the respondent self-identified as an intercalating student. The median age of student responders was 21 years old (interquartile range, 3 years), 69.1% (n=1,080) were women and 30.1% (n=471) were men, while 0.8% (n=13) preferred not to declare their gender. The 1,396 students (89.3%) studied in undergraduate-entry programs and 168 students (10.7%) were in the graduate entry. All year groups were represented (Fig. 1). The student responders represented 37 UK medical schools (Supplement 1).

### Independent study time

Before the pandemic, undergraduate-entry students reported spending more time studying independently than graduate-entry students (P<0.05). For all groups (men, women, undergraduates, and graduate students) the largest component of independent study time was allocated to university-provided information. This time spending, in combination with textbooks, represented approximately half of students’ study time. The other half of independent study time was dedicated to free or paid websites, podcasts, YouTube and other video resources, and online question banks. For all groups, the most used resources for independent study time were free websites and question banks.

When universities were shut to students because of the COVID-19 pandemic, students reported an increase in the mean weekly number of hours they spent studying independently irrespective of the route of entry (graduate-entry programs, P<0.001; undergraduate-entry programs, P<0.001). When comparing by gender, only women demonstrated a statistically significant increase (P<0.001 versus P=0.203). All cohorts except men reported an increase in the use of free websites and question banks (women, P<0.001; men, P=0.972; graduate-entry programs, P<0.05; undergraduate-entry programs, P<0.005), paid websites and question banks (women, P<0.001; men, P=0.531; graduate-entry programs, P<0.05; undergraduate-entry programs, P<0.001), and YouTube and other internet video resources (women, P<0.001; men, P=0.494; graduate-entry programs, P=0.005; undergraduate-entry programs, P<0.001). A significant increase was observed across all cohorts for apps, podcasts, and social media (women, P<0.001; men, P<0.05; graduate-entry programs, P<0.05; undergraduate-entry programs, P<0.001).

A consistent increase in resource use was not observed with regards to textbooks and journal articles, which showed a decrease in use by men students during COVID-19 (P<0.05). University information and personal notes, which also showed a decline in use by men students (P<0.001) as well as undergraduate-entry students overall (P<0.001).

**Fig. 2.**
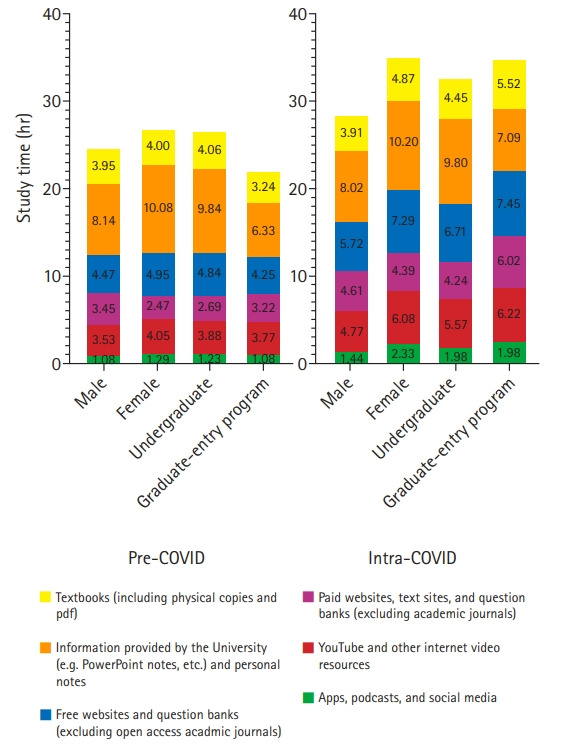
Comparison of the mean number of independent study hours using different study resources pre-coronavirus disease 2019 (COVID-19) versus during the COVID-19 lockdown for different student cohorts by gender (male vs. female) and course of study (undergraduates vs. graduate-entry program students).

Although undergraduate-entry students reported spending more time studying independently than graduate-entry students (P<0.05) before the pandemic, during COVID-19 lockdown, graduate-entry students surpassed the undergraduate program cohort in the number of hours spent studying independently (P<0.05). Although their independent study hours were similar before COVID-19 restrictions, women students demonstrated a much more significant increase in independent study time during COVID-19 than men (P<0.005) (Fig. 2).

### Frequency of use of study materials

**Fig. 3.**
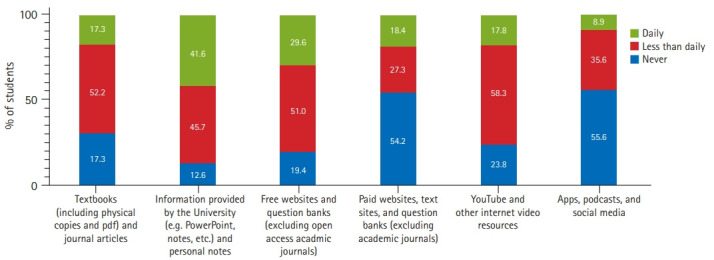
Frequency of use of different study resources within the 7 days prior to survey completion, conducted during the coronavirus disease 2019 lockdown period, as indicated by the percentage of student responders.

As depicted in Fig. 3, within the 7 days prior to survey completion, students reported using university information and personal notes most frequently (41.6% daily), followed by free websites and question banks (29.6% daily). Paid websites and question banks, as well as apps, podcasts, and social media were utilized least, with a majority of students reporting that they had not used these at all (54.2% and 55.6%, respectively).

### Motivational factors

**Fig. 4.**
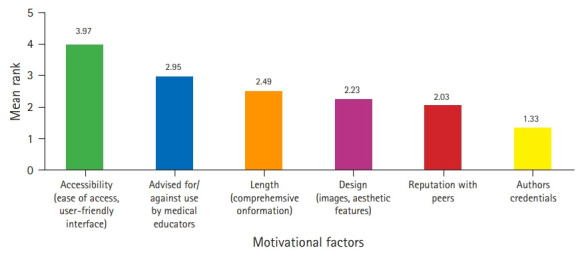
Factors impacting students’ use of independent study sources ranked from most important (6) to least important (1) (n=1,546) determined using the Friedman test (χ2=1,802.690, P<0.001).

**Fig. 5.**
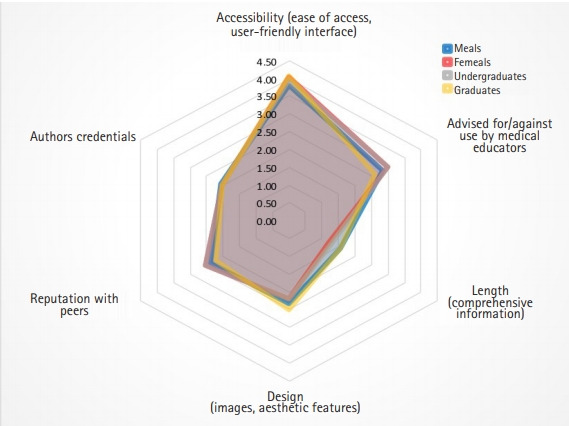
Factors impacting students’ use of independent study sources ranked from most important (6) to least important (1) for distinct student cohorts, including undergraduate-entry students (n=1,396) and graduate-entry program students (n=168), as well as female (n=1,080) and male (n=471) respondents as determined using the Friedman test.

Fig. 4 illustrates that the most important factor determining students’ opinion of FOAM resources was accessibility (μrank=3.97). The least important was author credentials (Fig. 4). These factors were ranked similarly across men and women as well as undergraduate and graduate-entry program students as indicated in Fig. 5, with the exception being design and length of resources, which were ranked as third and fourth most important for graduates, respectively.

### Awareness of FOAM

Despite students reporting a high usage of resources that can be characterized as FOAM, only 7.4% (n=116) had heard of the term, while the remainder were either unsure what FOAM was (7.2%, n=113) or had never heard of it previously (85.4%, n=1,335). Of those who provided a definition of FOAM, 86.6% of their statements concurred with the definition provided by Nickson and Cadogan [5].

### Changes in learning provisions during COVID-19

To explore whether the timing of a student’s response to the survey had affected their experience of learning provisions, we compared the first half of responses to the second half of responses (i.e., May versus June 2020) using the Cochran Q test. There was no statistically significant difference in learning provisions between these groups (P>0.05).

## Discussion

### Key results

University-provided materials were the single resource type to which students dedicated the most independent learning time. However, this comprised only a minority of total independent study hours. Instead, respondents reported dedicating half of their learning time to both free and paid websites and question banks, video resources such as YouTube, and more novel educational modalities such as apps, podcasts, and social media. During the COVID-19 pandemic, with clinical placements terminated for the overwhelming majority of participants, students increased the number of hours they spent studying independently.

### Interpretation

This increase in the independent study was driven by the increasing use of novel and online educational resources, a large proportion of which met the definition of FOAM [5]. Despite the shift of university materials online and towards formal online lectures, there was no consistently reported an increase in the use of university-provided materials. Similar trends were observed in the use of personal notes. Among male students and undergraduates, the amount of time dedicated to learning from such resources decreased. The amount of time allocated to sources beyond the university is in keeping with the current literature [9]. For example, a study of 522 medical students in 2011 reported that the vast majority believed that using sources other than those provided by their formal institutions was vital for passing examinations and that university-provided materials inadequately covered all of the material necessary [13].

A large study of Australian medical students also demonstrated an increasing reliance on question banks for study, many of which are subscription-only [9]. Previous studies have shown a correlation between the use of question books—the print forerunner to online question banks—and higher examination scores [14]. We found that overall students increased their use of paid websites during COVID-19 restrictions, but that a large subset (>50%) reported never using paid websites. This raises important questions about equitable access to learning materials as students move away from independent learning resources, such as textbooks, likely to be available via their educational institutions.

Together these findings paint an intriguing picture of students relying on university materials, increasingly supplemented by FOAM resources. Despite their growing reliance on FOAM resources, only 8% of participants had heard of the term “FOAM.” This phenomenon suggests that undergraduate medical students are accessing FOAM resources while being unaware of the philosophy underpinning the movement. This is reflected by students’ reported motivations for selecting particular learning resources: across all subgroups, students were most influenced by the accessibility and user-friendliness of resources. Students were least influenced by author credentials. This is in contrast to earlier studies, which found that peer recommendation was the most powerful motivating factor, in line with the collaborative and horizontal philosophy of FOAM [15]. We posit that as FOAM usage has become increasingly normalized among medical learners, there is declining recognition of its origins and traditional identity as a community of collaborators. Whether this changes as undergraduate learners become practicing physicians remains to be investigated, and therefore what this means for the FOAM movement is unclear.

### Limitations

The limitations of this study include lack of questionnaire validation and the use of snowball sampling as a means of survey distribution; while increasing the number of participants, this may have impacted the representativeness of the sample. Unequal representation of different year groups at each university could have affected sub-cohort analysis results. Additionally, no responses were gathered from 5 UK medical schools, and there was an unequal representation of participants from the remaining 37 medical schools. Data from each participant were collected cross-sectionally. This non-sequential survey limits the interpretation of the results. For example, if a student completed the study on a particular week in which there was no university material provided, the time spent studying independently may have been much higher than the following week when the material was provided. Additionally, results may have been influenced by the proximity of survey completion to examinations, since previous research has demonstrated that students’ resource use changes during exam season [9]. A potential solution to this may have been to capture data from each student longitudinally over a series of weeks.

### Generalizability

Due to the large and heterogeneous sample, the findings reported in this study can, to a great extent, be generalized to the broader population of UK medical students, especially those studying in undergraduate-entry programs, since the majority of our data was collected from this group. To a lesser extent, our findings could also potentially reflect the study behaviors of medical students studying in other countries, particularly courses run in developed and English-speaking nations (e.g., the United States, Canada, Australia), as evidenced by a recent study evaluating similar behaviors [9], as well as in other countries where medical schools follow a similar course structure to the MBBS (Bachelor of Medicine, Bachelor of Surgery) curriculum (e.g., India). However, generalizations at the international level should be made with caution, if at all, and must considerer factors influencing students’ ability to access SDL resources, including access to a quick and stable broadband connection, personal electronic devices, and financial affordability. In addition, language barriers associated with the use of such resources should be considered. One must also consider the unique nature of the year in which the data was collected. While students were asked to report pre-COVID study behaviors, novel stressors placed upon respondents may have had unforeseen effects on their self-reported answers. In order to evaluate the generalizability and reliability of our data, we recommend repeating the study once relative normality has returned to medical education provision within the United Kingdom.

### Suggestion

Universities should recognize students’ growing transition towards online study materials. However, if FOAM resources are to be effectively integrated into the medical curricula of the 2020s and beyond, there is a pressing need to better understand how students are using FOAM to supplement traditional teaching methods. This movement will likely involve large prospective trials evaluating current student behaviors and the effectiveness of interventions that incorporate FOAM resources. There is an additional need to ensure equitable access for all students to the resources. They need to access reputable and high-quality FOAM and other remote materials. There is alsoa pressing need for investigating medical students’ ability to select, appraise, and use educational materials independent of their educational institution.

### Conclusion

In this cross-sectional study of undergraduate medical students, we demonstrated a growing trend towards a relative increase in the use of learning resources independent of university provision. The COVID-19 pandemic has exaggerated these trends, while also contributing to the rise in the total time students have spent studying independently. The accessibility of such resources most strongly influences medical students’ decisions regarding resource choice, and students appear less influenced by factors such as peer recommendation. Alongside a lack of awareness of the FOAM movement, undergraduate medical students are interacting with online resources in a very different way from postgraduate populations. What this means for the future of the FOAM movement is unclear. What is clear, however, is that educators must appreciate the observed change in students’ independent learning behaviors. They must ensure equitable access to such materials and support students in making informed decisions about materials they use. Then,educators will be able to support students in choosing resources of sufficient quality to ensure that the students of today become the safe practitioners of tomorrow.

## Footnotes

**Data availability** Data files are available from Harvard Dataverse: https://doi.org/10.7910/DVN/78UW2J Dataset 1. Raw response data from 1,564 medical students in the United Kingdom to the survey questionnaire regarding study behaviors and Free Open Access Meducation.

## Supplementary materials

Supplementary files are available from Harvard Dataverse: https://doi.org/10.7910/DVN/78UW2J

Supplement 1. List of 32 medical schools of which students participated in the survey.

Supplement 2. Survey questionnaire regarding study behaviors and Free Open Access Meducation.

Supplement 3. Audio recording of the abstract.
