# Supplementary material for: Medical students’ pattern of self-directed learning prior to and during the coronavirus disease 2019 pandemic period and its implications for Free Open Access Meducation within the United Kingdom
Source: J Educ Eval Health Prof. 2021 Apr 6;18:5. doi: 10.3352/jeehp.2021.18.5 (PMC8144548; doi:10.3352/jeehp.2021.18.5)
Supplement: Supplementary file 3 — Supplement 2. Survey questionnaire regarding study behaviors and Free Open Access Meducation. [file jeehp-18-05-suppl2.docx]

Questionnaire (Questions were transcribed into electronic format.)

1. Sex

2. Age

3. Course and year of study

4. University currently attending

5. Prior to the coronavirus disease 2019 (COVID-19) pandemic, please estimate the number of hours per week you spent studying independently (i.e., outside of scheduled university teaching and clinical placements).

6. Please estimate the number of hours per week you currently spend studying independently (i.e., outside of scheduled university teaching and clinical placements).

7. Please estimate the % of time you spent prior to the COVID-19 pandemic, outside of scheduled university teaching and clinical placements, using each of the following sources of medical information for the purposes of learning:

a. Text books (inc. physical copies and .pdf) and journal articles

b. Information provided by the university (e.g., PowerPoints, notes, etc.) and personal notes

c. Free websites and question banks (excluding open access academic journals)

d. Paid websites text sites and question banks (excluding academic journals)

e. YouTube and other internet video resources

f. Apps, podcasts, and social media

8. Please estimate the % of time you currently spend, outside of scheduled university teaching and clinical placements, using each of the following sources of medical information for the purposes of learning:

a. Text books (inc. physical copies and .pdf) and journal articles

b. Information provided by the university (e.g., PowerPoints, notes, etc.) and personal notes

c. Free websites and question banks (excluding open access academic journals)

d. Paid websites text sites and question banks (excluding academic journals)

e. YouTube and other internet video resources

f. Apps, podcasts, and social media

9. In the past 7 days, how often did you use the following types of resources for the purpose of learning (will put box for daily, 3x, 2x, 1x, etc.)?

a. Text books (inc. physical copies and .pdf) and journal articles

b. Information provided by the University (e.g., PowerPoints, notes, etc.) and personal notes

c. Free websites and question banks (excluding open access academic journals)

d. Paid websites text sites and question banks (excluding academic journals)

e. YouTube and other internet video resources

f. Apps, podcasts, and social media

10. Please list your 5 **favorite** sources of medical information (state specific sources, e.g., names of books, names of websites, etc.)

11. How important are the following factors to your opinion of an internet source’s usefulness (please rank from 1–6, in order of importance):

a. Author credentials

b. Accessibility

c. Design (use of images or aesthetic features of the source)

d. Length

e. Reputation with peers

f. Advised for/against use by medical educators

12. Have you ever previously heard of the term FOAM (Free Online open Access Meducation/Medical Education)?

13. If so, please write a short definition (1 sentence) of your understanding.

14. Please select the following option which best describes how your education is being delivered during the COVID-19 pandemic:

a. Entirely self-directed learning

b. Remote delivery of some lectures/teaching sessions alongside self-directed

c. Continuing clinical placement

d. Paused studies

e. Other, please state
